# How antifoams act: a microgravity study

**DOI:** 10.1038/npjmgrav.2015.4

**Published:** 2015-05-27

**Authors:** Pavel Yazhgur, Dominique Langevin, Hervé Caps, Vincent Klein, Emmanuelle Rio, Anniina Salonen

**Affiliations:** 1 Laboratoire de Physique des Solides, Université Paris Sud—CNRS UMR, Orsay, France; 2 GRASP, Université de Liège, Liège, Belgium

## Abstract

Antifoams are widely used to control or to avoid foam production. In order to work, antifoam particles need to break foam films efficiently, which many antifoams do very well. However, once they have broken a film, to continue to be effective they need to be transported to the next film. We show, for the first time, that buoyancy has an important part in the transport of the antifoam particles. In microgravity, where buoyancy and gravitational drainage are strongly slowed down, diffusion leads to poor antifoam performance. The foam is stable for the duration of the experiment, whereas on Earth the foam starts to disappear immediately. Indeed, microgravity renders highly efficient antifoam practically useless.

Gas bubbles are often dispersed in water in the presence of stabilizing agents to form foams (see Figure 1) for applications including food, cosmetics, detergency, or oil recovery.^1^ There are also many instances where foams are unwanted and need to be avoided or destroyed, such as in washing or fermentation processes. Antifoam (AF) agents are therefore necessary.^2–4^ These agents are also used to control the amount of films in solid foams during the solidification of the precursor liquid foams. The elastic properties of the solid foams can thus be finely tuned.

AFs are divided into two families: fast and slow AFs depending on how quickly they destroy foams. Fast AFs make foam disappear within minutes, whereas slow AFs take tens of minutes.^2^ It is generally accepted that fast AFs act in films, whereas slow AFs flow out with capillary drainage and act after the Plateau borders (PBs) start draining. The general workings are relatively well described, but the microscopic mechanisms remain partly unclear, such as how slow AFs break the foam from the PBs.^3^ We have carried out foaming experiments both on Earth and in microgravity conditions with a fast AF. Microgravity conditions were obtained in parabolic flights. We used a surfactant solution of 4 g/l sodium dodecylsulfate that foams very well. Commercial antifoam (Silcolapse RG22, a gift from Bluestar Silicone) was used (0.7 wt% in the solution). This antifoam is an oil-in-water emulsion with a drop diameter *d*≈5 μm doped with silica particles. It is very efficient and can be classed as fast antifoam. We used a homemade foaming device^5^ consisting of a piston perforated by several holes moving back and forth at a frequency of 5 Hz in a cylinder containing the foaming liquid (height=5 cm, diameter=1.7 cm). The mean bubble radius is around 100 μm. The foam liquid fraction was varied between 10 and 70 vol%. A high-speed camera (Phantom Miro 310, at 200 frames/s) allowed following the foam generation and evolution.

In Figure 2, we see that the AF-free solutions (empty symbols) foam rapidly (inset) and are stable for the duration of the experiments both in 1 g and in μg (main graph). We also confirm that in 1 g, AF is effective at breaking the foam both during generation and afterward. In μg it is still effective during generation, although less so than in 1 g, but it is ineffective after generation.

We have previously shown that fast AFs are completely ineffective if no films are present.^5^ This would be the case if the gas fraction is below random close packing (64 vol%) so the bubbles remain spherical. In the inset of Figure 2, we plot the average time taken to generate the foam (the time to reach 90% of the final foam volume) at different volume fractions. The AF is ineffective at liquid fractions above 36%, in agreement with previous results.^5^ We also see that as the foam becomes drier, the AF becomes more effective during generation, but this depends on the gravity level.

It has been suggested that AFs are more effective during generation because bubble surfaces are not fully covered by surfactants.^6^ This should not depend on gravity and cannot be the sole reason why the AF is less efficient during generation in μg. Often, foaming methods are rather violent and the Froude number can be used to compare the role of gravity with that of inertia during generation Fr=*D*^2^/*g*, with *D*=5 cm the length of the piston and *υ*=5 Hz. This gives Fr=0.1 meaning that vigorous foam production is still rather weak in comparison with gravity. AF is thus more effective in 1 g than in μg, as more films are formed at the top of our sample in 1 g because of gravity-driven drainage.

The AF is almost inactive after generation in μg. We can imagine that straight after generation, the antifoam globules are evenly distributed throughout the foam in films, PBs and nodes. The films drain because of the capillary pressure, P, that sucks the liquid into the PBs.^7^ In these foams, *P* ≈3,000 Pa is much higher than hydrostatic pressure (at most 100 Pa), so film thinning proceeds in a very similar way in 1 g and in μg. The time to drain to around 5 μm is a fraction of seconds, after which most of the AF particles find themselves in PBs.

This means that, to continue to be effective the AF now have to make their way back toward the films to break them, as illustrated in Figure 1. In 1 g, the particles move within the PBs because of drainage, buoyancy (AF density not the same as water), and diffusion. We can estimate the time it takes to cross a PB (travel a distance of 10 μm) by means of each of these processes. A typical drainage velocity is 1 mm/s,^8^ which gives *t*_dr_ ≈1 s. The density of the droplets is around 1,050 kg/m^3^ from which we calculate a Stokes velocity of 40 μm/s and the corresponding time *t*_bu_ ≈20 s. Finally, the diffusion coefficient of the droplets is 10^−13^ m^2^/s, so *t*_di_ ≈1,000 s. In μg, drainage and buoyancy slow down by several orders of magnitude and they are not sufficiently fast to transport the AF toward the films to break the foam during the experimental time.

We highlight the importance of the transport step, which despite the extensive work carried out on AF mechanisms has been largely neglected (except in ref. 9). We show that buoyancy has a significant role in the transport of AF in the PBs. This result can also help explain why AFs on Earth are inefficient when the drops are too small for buoyancy to work efficiently.

## Figures and Tables

**Figure 1:**
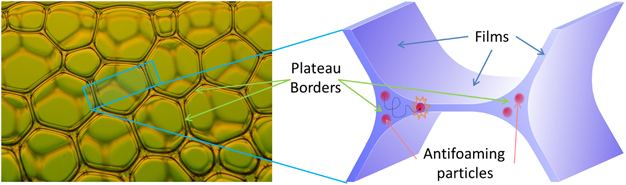
Photograph of foam and a schematic drawing of a film between two Plateau borders with enclosed antifoam particles.

**Figure 2:**
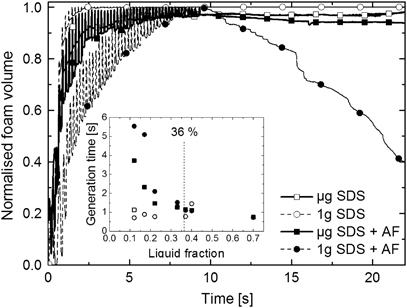
Normalized foam volume during the experiment with the generation time of the foam as a function of liquid fraction (inset). AF, antifoam; SDS, sodium dodecylsulfate.
